# Lactobacilli Expressing Broadly Neutralizing Nanobodies against HIV-1 as Potential Vectors for HIV-1 Prophylaxis?

**DOI:** 10.3390/vaccines8040758

**Published:** 2020-12-13

**Authors:** Sarah Kalusche, Kanika Vanshylla, Franziska Kleipass, Henning Gruell, Barbara Müller, Zhu Zeng, Kathrin Koch, Stefan Stein, Harold Marcotte, Florian Klein, Ursula Dietrich

**Affiliations:** 1 Georg-Speyer-Haus, Paul-Ehrlich-Straße 42-44, 60596 Frankfurt, Germany; kalusche@gsh.uni-frankfurt.de (S.K.); KochKathrin@prahs.com (K.K.); s.stein@gsh.uni-frankfurt.de (S.S.); 2 Laboratory of Experimental Immunology, Institute of Virology, Faculty of Medicine and University Hospital Cologne, University of Cologne, 50931 Cologne, Germany; kanika.vanshylla@uk-koeln.de (K.V.); franziska.kleipass@hhu.de (F.K.); henning.gruell@uk-koeln.de (H.G.); 3 Department of Infectious Diseases, Virology Centre for Integrative Infectious Diseases Research (CIID), University Hospital Heidelberg, 69120 Heidelberg, Germany; barbara.mueller@med.uni-heidelberg.de; 4 Department of Laboratory Medicine, Division of Clinical Immunology and Transfusion Medicine, Karolinska Institutet at Karolinska University Hospital Huddinge, 14186 Stockholm, Sweden; zengzhuydyxf@163.com

**Keywords:** nanobodies, VHH, prophylactic vaccine, neutralization, HIV-1, *Lactobacillus*, vector, humanized mouse model, passive immunization

## Abstract

In the absence of an active prophylactic vaccine against HIV-1, passively administered, broadly neutralizing antibodies (bnAbs) identified in some chronically infected persons were shown to prevent HIV-1 infection in animal models. However, passive administration of bnAbs may not be suited to prevent sexual HIV-1 transmission in high-risk cohorts, as a continuous high level of active bnAbs may be difficult to achieve at the primary site of sexual transmission, the human vagina with its acidic pH. Therefore, we used *Lactobacillus*, a natural commensal in the healthy vaginal microbiome, to express bn nanobodies (VHH) against HIV-1 that we reported previously. After demonstrating that recombinant VHHA6 expressed in *E. coli* was able to protect humanized mice from mucosal infection by HIV-1_Bal_, we expressed VHHA6 in a soluble or in a cell-wall-anchored form in *Lactobacillus rhamnosus* DSM14870. This strain is already clinically applied for treatment of bacterial vaginosis. Both forms of VHHA6 neutralized a set of primary epidemiologically relevant HIV-1 strains in vitro. Furthermore, VHHA6 was still active at an acidic pH. Thus, lactobacilli expressing bn VHH potentially represent an attractive vector for the passive immunization of women in cohorts at high risk of HIV-1 transmission.

## 1. Introduction

In 2019, 38 million people were living with HIV-1 worldwide [1]. Despite the increased availability of antiretroviral therapy (ART) for treatment as well as for prevention [2], the rate of new infections decreases slowly, with still 1.7 million persons newly infected by HIV-1 in 2019. Thus, a preventive vaccine against HIV-1 infection is still urgently needed. More than 30 years of intensive research towards an active HIV-1 vaccine have not yielded immunogens derived from the viral envelope protein (Env) that are able to induce broadly neutralizing antibodies (bnAbs) as an important correlate of protection from viral infections to date. The reasons for this are multifactorial, mostly Env-intrinsic, and were comprehensively summarized in a recent review [3]. However, bnAbs against HIV-1 could be identified in a subset of chronically infected patients several years after infection [4,5,6,7,8,9,10,11,12] and were shown to protect from viral challenge in animal models [7,13,14,15,16,17].

Some of these bnAbs have advanced to clinical trials assessing passive vaccination in therapeutic settings with the aim of reducing viremia in patients during treatment interruptions [4,6,10,18,19,20]. Although initially effective, viral escape mutants rapidly emerged in the patients upon antibody monotherapy. Thus, antibody combination therapy is required, as previously exemplified by ART [18,20,21]. Meanwhile some bnAbs also have advanced to clinical trials focusing on antibody-mediated protection (AMP) in cohorts at high risk of sexual HIV-1 transmission (NCT02716675 and NCT02568215) [22,23]. However, the large quantities (several grams per injection) and repeated administrations of bnAb required for effective protection in high-risk cohorts are difficult to achieve due to the high molecular weight and tetrameric structure of human antibodies whose formation requires complex cysteine bridges. Furthermore, prophylactic applications in the context of heterosexual transmission would require vaginal application of bnAbs, e.g., as a gel-formulated microbicide, and stability and functionality at acidic pH must be ensured [24]. Therefore, in view of HIV-1 prevention strategies, nanobodies or VHHs, which correspond to the smallest antigen-recognizing domains of heavy-chain-only antibodies from camelids (recently reviewed in [25,26]), are a promising alternative for clinical applications. Besides their small molecular mass (15 kDa), high solubility and stability, VHHs are highly homologous to human VH3 sequences. Of particular interest in the context of HIV-1 are their usually long complementary determining region 3 (CDR3), which is a characteristic feature of many bnAbs against HIV-1 [27]. The first VHHs with broad neutralizing capacity against primary HIV-1 strains were described by the group of R. Weiss [28].

We recently selected a set of potent and bn VHHs from dromedaries immunized with soluble trimeric Env constructs from HIV-1 subtype C, the most prevalent HIV-1 subtype worldwide [29]. These VHHs neutralize primary HIV-1 strains of different subtypes with high potency, and therefore are promising candidates for further development towards clinical applications for HIV-1 prevention. As the small size of VHHs (15 kDa) comes along with a short half-life, vector-mediated expression is an option to maintain therapeutically active protein concentrations over time. With the final aim of preventing sexual transmissions of HIV-1 in high-risk cohorts, we chose *Lactobacillus* vectors for several reasons: (i) Lactobacilli are GRAM-positive bacteria naturally occurring in the healthy human vagina and are generally regarded as safe (GRAS) by the FDA. (ii) They protect against human pathogens by multiple mechanisms such as competitive inhibition, secretion of bacteriocins and generation of a low vaginal pH through the secretion of lactic acid [30,31]. Vice versa, the absence of vaginal *Lactobacillus* species is associated with bacterial vaginosis, genital inflammation, immune activation and increased risk of HIV-1 acquisition [32,33]. (iii) Besides having antiviral properties per se, lactobacilli can be engineered to express heterologous antiviral molecules secreted into the media or covalently anchored to their surface [34,35,36,37,38,39,40,41,42,43,44].

Here, we express our most potent HIV-neutralizing nanobody, VHHA6, in *Lactobacillus rhamnosus* DSM 14870, a strain, which has been well characterized and which is already in clinical use as a constituent of vaginal capsules (EcoVag, Bifodan A/S, Hundested, Denmark) for treatment of bacterial vaginosis. Furthermore, this strain was previously shown to effectively colonize the human vagina [45,46,47,48]. After showing protection from mucosal HIV-1 infection in a humanized mouse model by soluble VHHA6 produced in *E. coli* in a proof-of-concept study, we expressed VHHA6 from recombinant *L. rhamnosus* DSM 14870, both in a soluble and in a surface covalently anchored form. Both forms neutralize primary HIV-1 strains of different subtypes in vitro and binding still occurred at acidic pH as typically found in the vaginal milieu. Thus, *Lactobacillus rhamnosus* DSM 14870 expressing bn nanobodies may represent a promising vector for HIV-1 prophylaxis in women at high risk of sexual HIV-1 acquisition.

## 2. Materials and Methods

### 2.1. Expression of Recombinant Soluble optC gp140 (optC.664) SOSIP Proteins

OptC gp140 SOSIP constructs were previously generated from a codon-optimized HIV-1 subtype C *env* consensus sequence [29]. NL4-3 SOSIP was created similarly in the Dietrich group by a former PhD student (Svenja Weiß, unpublished). Second generation SOSIP (optC.664) was generated by introducing a stop codon after the ectodomain sequence of gp41 via PCR [49]. A total of 70 µg *env* DNA and 35 µg furin DNA (D. von Laer, Georg-Speyer-Haus, Frankfurt, Germany) were used to transiently transfect 2 × 10^7^ HEK293T cells (ATCC^®^ CRL-11268™) using 210 µL polyethylenimine (1 mg/mL, Sigma-Aldrich, Darmstadt, Germany). Supernatants were harvested after 48 h, and gp140 SOSIP proteins were purified via lectin (*Galanthus nivalis*, Sigma-Aldrich, Germany) affinity chromatography (elution with 10 mL 0.5 M Methyl-α-D-mannopyranoside (Sigma-Aldrich, Darmstadt, Germany)) and buffer exchanged to PBS (PAA Laboratories GmbH, Pasching, Austria) using Amicon^®^ Ultra-4 centrifugal filters (100 kDa cut-off; Merck Millipore, Burlington, MA, USA). For ELISA, SOSIP proteins were not further purified via SEC or AEC.

### 2.2. Expression of VHH Proteins in E. coli

VHHs, which were previously cloned into the pCAD51 expression vector (contributed by R. Weiss, University College, London [29]), were expressed from *E. coli* HB2151 cells (BioScience, Cambridge, UK). For VHH production, an overnight culture diluted 1:100 was grown in LB-Amp/0.1% glucose until an OD_600_ of 0.5–0.7 was reached. VHH expression was induced with IPTG (final concentration 1 mM). Bacterial cultures were incubated at 28 °C and 180 rpm overnight. After centrifugation (20 min, 4750RCF, 4 °C), cell pellets were resuspended in 5 mL TES buffer/g pellet (0.2 M Tris, 0.65 mM EDTA, 0.5 M sucrose, pH 8) and incubated on ice for one hour. Subsequently, 10 mL TES/4 buffer/g pellet (TES buffer diluted 1:4 in dH_2_O) were added and incubated on ice for further 45 min. After centrifugation at 10,000× *g* for 30 min at 4 °C, the filtrated VHH-containing supernatants were applied to 1 mL HisTrap^TM^ HP columns (GE Healthcare, Amersham, UK) using ÄKTA FPLC P920 (GE Healthcare Europe GmbH, Frankfurt, Germany). VHHs were eluted with 80% elution buffer (50 mM Na_2_HPO_4_/NaH_2_PO_4_, 300 mM NaCl, 250 mM Imidazol), following buffer exchange to PBS using Amicon^®^ Ultra-4 centrifugal filters (10 kDa cut-off; Merck Millipore, Burlington, USA). Protein concentration was determined with the Pierce™ BCA Protein Assay Kit (Thermo Fisher Scientific, Dreieich, Germany) according to manufacturer’s instructions.

### 2.3. Cloning and Expression of VHH Proteins in L. rhamnosus

For expression in *Lactobacillus rhamnosus* DSM14870 (Bifodan A/S, Hundested, Denmark), VHHA6 was cloned into plasmids originating from the pIAV7 vector [50]. For soluble expression, we used pAF100 containing a C-tag (also known as EPEA-tag), whereas, for the surface-anchored version, we used pAF900, which contains an E-tag [42]. Covalent surface-binding was achieved by linking the VHH gene to the *prtP* anchor region-encoding gene. Both plasmids contain an erythromycin resistance gene. *L. rhamnosus* cultures were grown in MRS Broth media (Merck Millipore, Burlington, USA) with 5 µg/mL erythromycin (Carl Roth GmbH, Karlsruhe, Germany) at 37 °C without shaking.

The expression of VHHA6 from *L. rhamnosus* pAF100-VHHA6 (C-tag) and *L. rhamnosus* pAF900-VHH (E-tag) was confirmed by Western blot. The bacterial cultures were spun down, when OD_600_ reached 1.0 and the supernatant and bacterial pellets were separated for protein extraction. The supernatant was filter-sterilized and the pH adjusted to 7.0. Then, 100 μL of supernatant was mixed with an equal volume of 2× Laemmli buffer (Bio-Rad, Dreieich, Germany) and boiled for 5 min. The bacterial pellets from 1 mL cultures were washed twice with PBS, resuspended in 100 μL 2× Laemmli sample buffer and boiled for 10 min. The cell debris was removed by centrifugation at 16,100× *g* and the supernatant, denoted as cell extract, was retained. The supernatant and cell extract of *L. rhamnosus* pIAV7 were used as a negative control.

A total of 20 μL of the supernatant or cell extract sample were run on an SDS-PAGE gel and transferred onto a nitrocellulose membrane (Hybond-ECL, GE Healthcare, Amersham, UK). The secreted VHHA6 fused to a C-tag was detected using biotinylated VHH anti-C-tag antibody (1 µg/mL, Thermo Fisher Scientific, Dreieich, Germany) and horseradish peroxidase (HRP)-conjugated streptavidin (1/2000, Becton, Dickinson and Company, Franklin Lakes, NJ, USA) diluted in 1% milk. The anchored VHH fused to an E-tag was detected using mouse monoclonal anti-E-tag antibody (1 µg/mL, 1.5 h, Phadia AB, Uppsala, Sweden) and HRP-conjugated goat anti-mouse antibody (1/1000, 1.5 h, Dako A/S, Glostrup, Denmark). The membranes were developed using ECL chemiluminescent substrate (GE Healthcare, Amersham, UK).

Soluble VHH fused to the C-tag was purified from the culture supernatant via Capture Select C-tagXL affinity matrix (ThermoFisher Scientific, Dreieich, Germany). Briefly, *L. rhamnosus* pAF100-VHHA6 was grown in a 200 mL volume of modified MRS broth (without beef extract) until an OD_600_ of 1.0. The culture supernatant was filter-sterilized, adjusted to pH 7.3 and passed through a 5 mL pre-packed column. The VHH was eluted with 20 mM Tris, 2.0 M MgCl_2_, pH 7.0, and dialyzed against PBS, pH 7.4 overnight at 4 °C using dialysis tubes (Molecular Weigh cut off 3KD, Spectrum Labs Spectra/Por, Thermo Fisher Scientific, Dreieich, Germany).

### 2.4. Expression of Recombinant VHH-Fc Proteins

Recombinant VHHA6 cDNA was cloned into the pCMX expression vector (contributed by M. Hust, TU Braunschweig), which encodes an optimized human IgG1 Fc domain. A total of 20 µg CMX VHH-Fc plasmid DNA and 60 µL PEI (1 mg/mL) were used to transiently transfect 5 × 10^6^ HEK293T cells. Supernatant was harvested after 48 h. Recombinant VHH-Fc proteins were purified using Pierce™ Protein A Agarose columns (Thermo Fisher Scientific, Dreieich, Germany) according to manufacturer’s instructions. Eluted VHH-Fc proteins were concentrated and buffer exchanged to PBS, as described for VHHs (Section 2.2).

### 2.5. Acidic pH ELISA

ELISA plates were coated with optC gp140 SOSIP Env (0.2 µg SOISP/well; Greiner Bio-One, Kremsmünster, Austria) and blocked with 250 µL PBS containing 5% milk powder (Sigma-Aldrich, Darmstadt, Germany). Afterwards, 250 ng of the different VHHs were added for one hour in 100 μL PBS with different pH (7.4, 4.2 or 3.7). PBS without VHH served as negative control and mAb b12 (NIH AIDS Research & Reference Reagent Program, Division of AIDS, NIAID, Germantown, USA) as full antibody for comparison. Between all steps, ELISA plates were washed three times with PBS supplemented with 0.05% Tween-20 (Sigma-Aldrich, Darmstadt, Germany). Bound VHHs were detected by addition of mouse anti-c-myc antibody (AbD Serotec, USA) followed by anti-mouse-IgG-HRP (GE Healthcare, Amersham, UK). MAb12 was detected via anti-human Fc-HRP (Jackson ImmunoResearch, Cambridgeshire, UK). As control for SOSIP gp140 Env epitope integrity, the SOSIP was pretreated for 1 h with acidic PBS (pH 4.2 and 3.7), before 100 ng of VHHs were added to the wells for one hour in 100 μL PBS (pH 7.4).

### 2.6. Neutralization Assays for Soluble Nanobodies

Neutralization capacity of VHHs was determined in a standard TZM-bl assay [51]. All HIV-1 Env pseudotyped viruses used in the experiments were obtained from the HIV Specimen Cryorepository (H. von Briesen, Fraunhofer IBMT, St. Ingbert, Germany).

Purified VHHs were assayed in duplicates for neutralization in three-fold serial dilutions starting with a concentration of 50 µg/mL. A total of 50 µL pseudovirus (15,000 RLU (relative luminescence units)) were added to the diluted VHHs and incubated at 37 °C for 60 min. 1 × 10^4^ TZM-bl cells (NIH AIDS Research & Reference Reagent Program, Division of AIDS, NIAID, Germantown, USA) were applied per well (containing 12.5 µg/mL DEAE-dextran, Sigma-Aldrich, Darmstadt, Germany) and grown for 48 h. Cells were lysed in harvest buffer (1.25 mL 1 M MES-Tris pH 7.8 (Carl Roth GmbH, Karlsruhe, Germany), 25 µL 1 M DTT, 2.5 mL glycerol, 250 µL 10% Triton X-100 (all Sigma-Aldrich, Darmstadt, Germany) in 25 mL dH_2_O) for 10 min at room temperature and frozen at −80 °C overnight.

Luminescence was measured by blending 50 µL luciferin solution (25 µL luciferase buffer (6.25 mL 1 M MES-Tris, 1.25 mL 1 M MgCl_2_ (Carl Roth GmbH, Karlsruhe, Germany), 120 mg ATP (Sigma-Aldrich, Darmstadt, Germany), 42.5 mL dH_2_0) and 25 µL luciferin (10 mg luciferin (Promega, Walldorf, Germany), 36 mL 5 mM K_2_HPO_4_/KH_2_PO_4_ (Carl Roth GmbH, Karlsruhe, Germany), pH 7.8) with 60 µL thawed cells using a Lumistar Galaxy plate reader (BMG LABTECH GmbH, Ortenberg, Germany). Neutralization was determined as the reduction in relative luminescence units (RLU) in sample wells compared to RLU in virus control wells after subtraction of background luminescence. The 50% inhibitory concentration (IC_50_) was defined as the reciprocal VHH dilution resulting in 50% reduction in RLU compared to virus control wells. IC_50_ values were determined using Prism software (GraphPad Software, Inc., San Diego, CA, USA).

### 2.7. Infectivity Depletion Assay for Cell Surface-Anchored Nanobodies

*L. rhamnosus* with plasmids pAF900-VHHA6 and pIAV7 (negative control) were cultured in MRS Broth media (Sigma-Aldrich, Darmstadt, Germany) containing 5 µg/mL erythromycin at 37 °C without shaking, until OD_600_ = 1 was reached. Bacteria were washed with PBS and irradiated with 120,000 µJoules for 2 min using a UV-Transilluminator (GelDoc 2000, Bio-Rad, Dreieich, Germany). After resuspension in cell culture media with 0.05 M Methyl-α-D-mannopyranoside (Sigma-Aldrich, Darmstadt, Germany), serial dilutions were performed in duplicates starting with an OD_600_ = 5 (150 µL end volume). 50 µL pseudovirus (15,000 RLU) were added to the diluted bacterial cells and incubated at 37 °C for 60 min. Plates were centrifuged at 2500 RCF for 10 min (MEGAFUGE 1.0R, Heraeus Instruments, Hanau, Germany) at 4 °C and the supernatant containing depleted (unbound) pseudovirus was added to 1 × 10^4^ freshly trypsinized TZM-bl cells in 50 μL growth medium supplemented with 12.5 μg/mL DEAE-Dextran. Cells were grown for 48 h before being lysed in harvest buffer for 10 min at room temperature and frozen at −80 °C overnight.

Luminescence was measured as described in 2.6. Neutralization was determined as the reduction in RLU in sample wells compared to RLU in virus control wells after the subtraction of background luminescence. The 50% inhibitory optical density (IOD_50_) was defined as the reciprocal bacterial dilution resulting in 50% reduction in RLU compared to virus control wells. IOD_50_ values were determined using Prism software.

### 2.8. Production of pCHIV Pseudovirus

For the production of eGFP labeled pseudovirus, HIV-1-derived plasmids pCHIV and pCHIVeGFP [52], which express all proteins from HIV-1NL4-3 except for Nef, were used. Transfection of these plasmids yields non-infectious eGFP labeled particles. 5–6 × 10^6^ HEK293T cells were seeded into 10 cm tissue culture dishes one day prior to transfection. Shortly before transfection, medium was exchanged with DMEM without FCS supplemented with chloroquin (final concentration 25 µM, Sigma-Aldrich, Darmstadt, Germany). The transfection mixture contained 10 μg pCHIV and 10 μg pCHIVeGFP, 50 μL 2.5 M CaCl_2_ (Carl Roth GmbH, Karlsruhe, Germany) and 450 μL H_2_O. Derivatives of these plasmids carrying a 2 bp insertion at the NdeI site, resulting in a frameshift in the viral *env* gene were used to prepare ΔEnv control particles. For transfection, the mixture was added to 500 μL 0.2 M HEPES buffer, pH 7.05 (Sigma-Aldrich, Darmstadt, Germany) and incubated for 20 min at room temperature. The transfection mixture was added to the prepared 293T cells and cultured for 6 h before medium exchange. The supernatant was collected after 12 and 36 h, then filtrated and concentrated by overnight centrifugation at 6000 rpm and 4 °C. The supernatant was discarded, and the pellet was resolved in 100 µL of PBS. The particle concentration was quantified by p24 ELISA (Innogenetics, Gent, Belgium).

### 2.9. Analysis of Lactobacilli by Flow Cytometry

*L. rhamnosus* with plasmids pAF900-VHHA6 and pIAV7 were cultured as described in 2.3., until OD_600_ reached 1. For flow cytometry, bacteria were washed with PBS and 3 × 10^7^ cells were transferred into a 2 mL tube. A total of 2 µL rabbit anti-E tag antibody (Abcam, Cambridge, UK) was added for 30 min on ice. Cells were washed with 1 mL PBS, before adding 2 µL of the goat anti-rabbit IgG-BV421 (Becton, Dickinson and Company, Holdrege, USA) for 30 min in the dark. Following a washing step, cells were fixed with 400 µL 4% formaldehyde (Sigma-Aldrich, Darmstadt, Germany) at room temperature. A total of 5 µL Thiazole Orange (Becton, Dickinson and Company, Holdrege, UK) were added before analysis of bacterial cells with BD FACSCanto^TM^ II.

To analyze binding of VHHA6 on the surface of *L. rhamnosus* to native Env protein incorporated in the pCHIV pseudovirus, 3 × 10^7^ bacterial cells were transferred into a 2 mL tube in PBS with 2% BSA (Serva, Heidelberg, Germany) and 0.05 M Methyl-α-D-mannopyranoside. Supernatant (2.5–20 µL) containing eGFP-labeled pseudovirus was added to the cells for 4–5 h at 4 °C. Following washing, cells were fixated and analyzed by flow cytometry (BD FACSCanto^TM^ II).

### 2.10. p24 ELISA

Since virus titration was not possible for pCHIV without incorporated Env, p24 content was determined to correlate supernatants containing pseudovirus with and without Env. Therefore, the p24 ELISA kit (Innogenetics, Gent, Belgium) was used according to the instructions from the manufacturer. The P24 antigen content of NP40-inactivated virus supernatants was calculated in relation to the supplied standard.

### 2.11. Mouse Experiments

To determine the in vivo pharmacokinetic profile of VHHA6 purified from *E.coli* culture supernatants and the heavy-chain-only antibody A6-Fc in comparison to 3BNC117, NOD RAG1^−/−^ IL2Rγ^NULL^ (NRG) mice were intravenously injected with 500 µg of protein (tail vein, three mice per group). The half maximal inhibitory dose (ID_50_) of serum dilutions was determined against HIV-1 Tro.11 and MLV control pseudoviruses in the TZM-bl assay. Blood samples were taken over 120 h to determine the respective nanobody/antibody concentrations in the mice sera. These were calculated based on the ID_50_ and IC_50_ values determined against the Tier 2 HIV-1 Tro.11 pseudovirus in the TZM-bl assay according to the formula

(1) $$\mathrm{concentration}\;\left[\frac{\text{µ}g}{\mathrm{mL}}\right]=\;{\mathrm{IC}}_{50}\;\left[\frac{\text{µ}g}{\mathrm{mL}}\right]{\times \;{\mathrm{ID}}_{50}}_{\;}$$

In vitro neutralization activity of VHHA6 and control VHH6G2 (selected against a bacterial transporter protein by Dr. E. Geertsma, Max Planck Institute of Molecular Cell Biology and Genetics, Dresden, Germany, unpublished) against HIV-1 NL4-3_BAL_ infectious molecular clone (IMC, *env* from HIV-1_BAL_ in pNL4-3, here referred to as HIV-1_BAL_ [53]) was evaluated using TZM-bl neutralization assay. To determine the intrarectal (i.r.) mucosal transmission efficiency of HIV-1_BAL_, CD34T+ mice (4 mice) [54] were challenged on two consecutive days with 1.2 × 10^5^ TCID50 HIV-1_BAL_ by directly pipetting 40 μL virus at the rectal opening without causing injury.

For evaluating the prophylactic effect of VHHA6 in vivo, CD34T+ mice were intrarectally challenged on three consecutive days with 6.2 × 10^4^ TCID_50_ HIV-1_BAL_. Prior to i.r. application, the virus was incubated for 15 min at room temperature with 50 µg VHHA6 (nine mice), 50 µg VHH 6G2 (6 mice) or an equivalent volume of PBS (two mice; an additional four mice were injected in a previous experiment). For measuring viremia in mice, HIV-1 RNA was isolated from plasma using the QIAcube (Qiagen, Hilden, Germany) and the QIAamp MinElute Virus Spin Kit (Qiagen, Hilden, Germany) with an additional DNaseI (Qiagen, Hilden, Germany) digestion step. Viral loads were determined as previously described [21] by quantitative real-time PCR using *gag-*specific primers 6F 5′-CATGTTTTCAGCATTATCAGAAGGA-3′ and 84R 5′-TGCTTGATGTCCCCCCACT-3′ and *gag*-specific probe 56-FAM/CCACCCCACAAGATTTAAACACCATGCTAA/ZenDQ [55]. qPCR was performed on the QuantStudio 5 system (Thermo Fisher, Carlsbad, USA) using the TaqMan RNA-to-CT 1-Step Kit (Thermo Fisher, Carlsbad, USA). An HIV-1 standard derived by super-infection of SupT1-R5, with a pre-determined copy number, measured using the Cobas 6800 HIV-1 kit (Roche, Mannheim, Germany), was included in every RNA isolation/qPCR run. The quantification limit of the qPCR was determined to be 384 HIV-1 RNA copies/mL. Samples with viral load values below the detection limit or completely negative samples were assigned values between 100 and 300 for plotting graphs in GraphPad Prism 7.

## 3. Results

### 3.1. Functionality of Nanobody VHHA6 Expressed from E. coli

As proof of concept for in vivo applications of nanobodies in terms of HIV prevention, we first analyzed one of our best broadly neutralizing nanobodies, VHHA6 [29], with respect to essential parameters like in vivo half-life and the capacity to prevent HIV-1 infection in humanized NRG mice.

#### 3.1.1. Determination of In Vivo Half-Life of VHHA6 in Sera of Mice

To evaluate the pharmacokinetic profile of VHHA6 in comparison to its heavy-chain-only variant VHHA6-Fc and a full-length antibody, we intravenously injected 500 µg of VHHA6, VHHA6-Fc or bnAb 3BNC117 into NRG mice. Longitudinal serum concentrations of the individual constructs were derived from the serum ID_50_s (Supplementary Figure S1a) and monoclonal 50% inhibitory concentrations (IC_50_) determined in the TZM-bl cell neutralization assay. Forty-five minutes after injection, protein levels were highest for all three constructs with little difference between nanobody VHHA6 and VHHA6-Fc (Supplementary Figure S1b). At this timepoint, the concentration of VHHA6 in sera was 7.6 µg/mL (VHHA6-Fc: 19 µg/mL, 3BNC117: 135 µg/mL). However, as expected, protein concentration decreased over time depending on the size of the constructs. Whereas the small VHHA6 nanobody (15 kDa) was not detectable anymore at 6 h after injection, the respective Fc fusion construct (80 kDa) and the control mAb (150 kDa) were still detectable after 120 h (1.3 and 14.5 µg/mL, respectively).

#### 3.1.2. Soluble VHHA6 Blocks Infection of Humanized Mice by HIV-1

**Figure 1:**
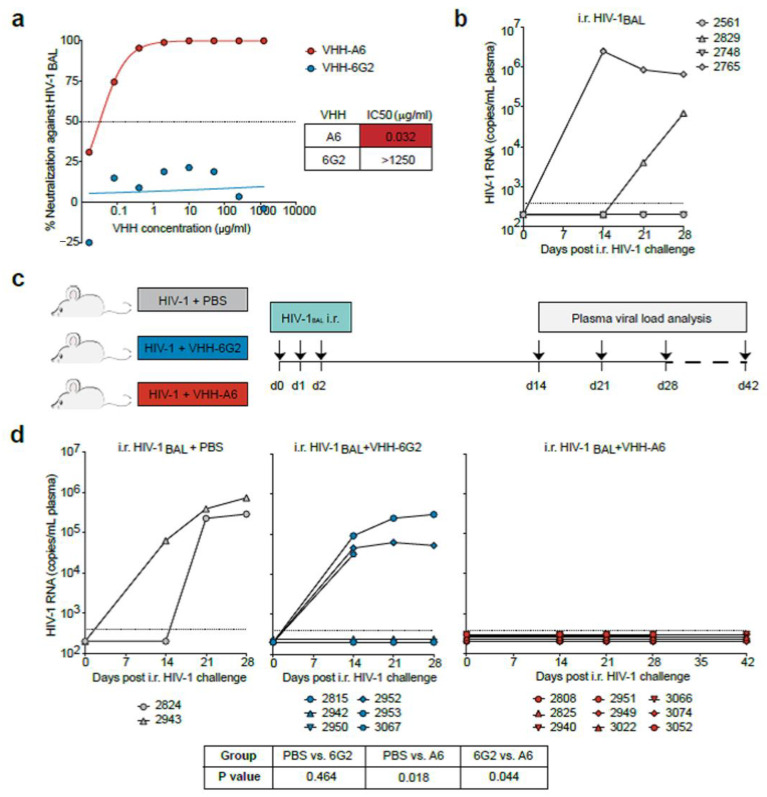
VHHA6-mediated prevention of mucosal HIV-1 infection in humanized mice. (**a**) Neutralizing activity of anti-HIV-1 VHHA6 and control VHH6G2 against the HIV-1_BAL_ infectious molecular clone (IMC) using TZM-bl cells. Dotted line represents the IC_50_. (**b**) Analysis of HIV-1 RNA levels in CD34T+ mice (N = 4) following high-dose intra-rectal (i.r.) HIV-1_BAL_ challenges on two consecutive days. Dotted line represents the quantification limit of 384 HIV-1 RNA copies/mL of the qPCR assay. (**c**) Schematic representation of the experimental design used to test VHHA6–mediated HIV-1 prevention. CD34T+ humanized mice were given high-dose i.r. challenges with HIV-1_BAL_ mixed with either PBS (N = 2), 50 µg control VHH6G2 (N = 6) or 50 µg anti-HIV-1 VHHA6 (N = 9) on three consecutive days and plasma viremia was tracked over time. (**d**) Analysis of HIV-1 RNA levels in the different CD34T+ mouse groups as described in (**c**). Dotted line represents the quantification limit of 384 HIV-1 RNA copies/mL of the qPCR assay. Statistical analysis in (**d**) was done using Fisher’s exact test.

We then analyzed the capacity of nanobody VHHA6 to block HIV-1 infection in humanized mice (Figure 1). We used an infectious molecular clone of HIV-1 containing the BAL envelope sequence and first confirmed the sensitivity of the HIV-1_BAL_ virus to neutralization by VHHA6 (Figure 1a). The in vivo mucosal transmission efficiency of the HIV-1_BAL_ virus clone was tested to be about 50% in CD34T+ humanized mice (Figure 1b). To analyze A6-mediated HIV-1 prevention in an independent experiment including appropriate controls, CD34T+ mice were intrarectally (i.r.) challenged on three consecutive days with 6.2 × 10^4^ TCID_50_ HIV-1_BAL_. Prior to i.r. application, the virus was incubated either with 50 µg VHHA6 (nine mice), 50 µg control VHH6G2 (six mice) or with an equivalent volume of PBS (two mice) for 15 min at room temperature (Figure 1c). Plasma HIV-1 RNA was measured for up to 6 weeks by quantitative real time PCR. Whereas five of eight control mice (62.5%), which received either PBS or the control VHH 6G2 (Figure 1d, left and middle panel), were infected by HIV-1_BAL_ after three consecutive i.r. challenges, 9/9 mice receiving high-dose HIV-1_BAL_ in conjunction with VHHA6 three times were protected from infection (Figure 1d, right panel).

These results prove the capacity of nanobody VHHA6 to block infection by HIV-1_Bal_ in a humanized mouse model upon i.r. challenge. However, due to the short half-life of nanobodies, vector-mediated continuous production will be needed in view of its development towards a prophylactic passive vaccine.

#### 3.1.3. Nanobodies Retain Functionality at Acidic pH

**Figure 2:**
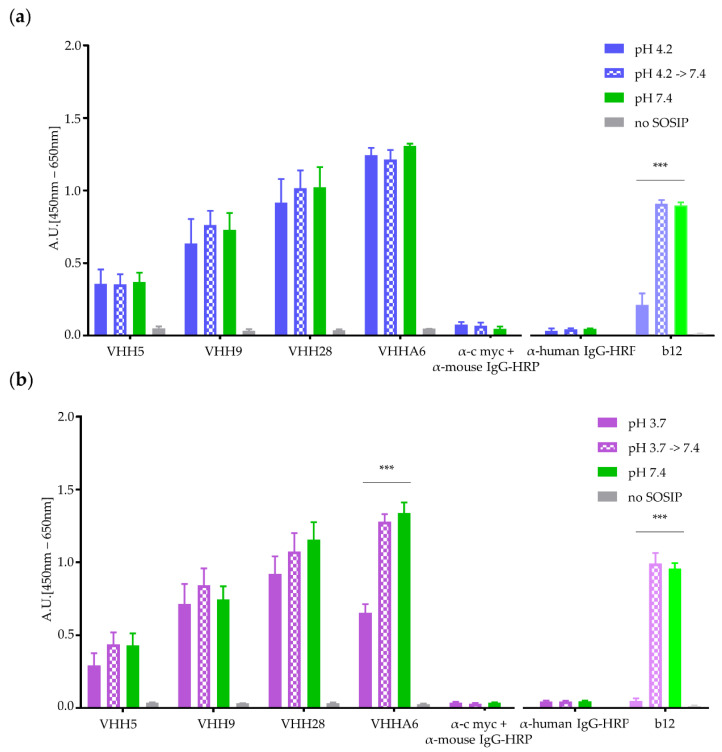
Binding of purified VHHs to optC.664 gp140 SOSIP at acidic pH. Binding experiments were performed using 250 ng of VHHs 5, 9, 28 and A6 [29] and 200 ng immobilized optC.664 gp140 SOSIP at pH 4.2 (**a**) and pH 3.7 (**b**). Binding of VHHs was detected by ELISA via their C-terminal myc-tag. Binding efficiency of VHHs at acidic pH (blue/pink filled bars, respectively) was compared to binding at pH 7.4 (green bars). Full human mAb b12 was included for comparison (detection with α-human IgG-HRP). SOSIP pretreated at acidic pH for 1 h was used as control for antigen integrity (spotted bars). α-c myc and α-human IgG antibodies represent controls without addition of VHHs. Error bars represent SEM of duplicates from three experiments. Statistical differences in absorption between pH 7.4 and acidic pH were determined using t-test. ***: *p* < 0.001, **: *p* < 0.01; *: *p* < 0.05.

For prophylactic application of HIV-1 neutralizing nanobodies in the human vagina, the pH stability of the nanobodies must be ensured. The vaginal milieu is characterized by an acidic pH between 4 and 4.5 in healthy women [30]. To analyze the functionality of nanobodies in an acidic environment around pH 4.0, we tested VHHA6 and other previously selected nanobodies targeting the CD4-binding site in Env (VHH 28, VHH 9 and VHH 5 [29]) for binding efficiency to optC.664 gp140 SOSIP at acidic pH in ELISA (Figure 2).

Binding was performed at pH 4.2 and pH 3.7 and compared to binding of VHHs at neutral pH (7.4) to gain insight into nanobody functionality around the lowest pH (4.0) found in the human vagina. Binding of mAb b12 at the respective pH was analyzed for comparison. Whereas at pH 4.2 (Figure 2a), binding of nanobodies was not impaired, binding of control mAb b12 was strongly reduced. At pH 3.7 (Figure 2b), binding of mAb b12 was already completely abolished. In contrast, all nanobodies were still able to bind to their target, although binding was reduced for VHH 5 and A6. The reduced binding efficiency was not due to acidic effects directly on the Env target protein, as preincubation of optC.664 gp140 SOSIP for 1 h at acidic pH did not affect binding of mAb b12 and nanobodies at neutral pH (hatched bars in Figure 2).

After proving binding of nanobodies to HIV-1 Env at acidic pH, as present in the human vagina, we decided to use *Lactobacillus rhamnosus* DSM 14870 as a suitable expression vector in view of prophylactic applications against sexual HIV-1 transmissions. The major reason for choosing this vector was that this strain is a natural commensal in the human healthy vagina and is already a constituent of vaginal capsules (EcoVag, Bifodan A/S, Hundested, Denmark) for treatment of bacterial vaginosis. This approach would ensure the safe and continuous supply of HIV-1-neutralizing nanobodies, despite their short half-life

### 3.2. Functionality of Nanobody VHHA6 Expressed from L. rhamnosus DSM 14870

#### 3.2.1. Soluble VHHA6 Expressed from *L. rhamnosus* Retains Neutralizing Activity against HIV-1

**Figure 3:**
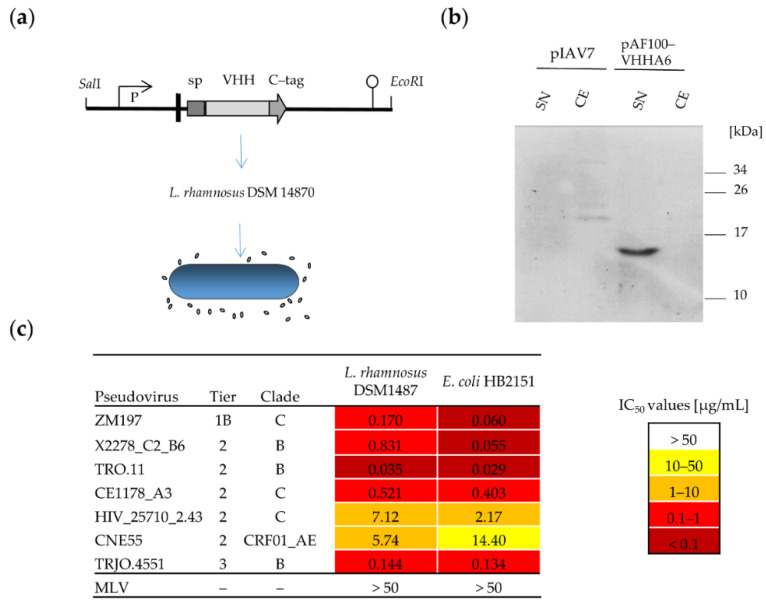
Expression of secreted VHHA6 from *Lactobacillus rhamnosus* DSM 14870 pAF100-VHHA6. (**a**) The expression cassette was cloned into the pIAV7 plasmid [50] between *SalI* and *EcoRI* restriction sites generating the plasmid pAF100 VHHA6. The expression cassette of VHHA6 is controlled by the *apf* promotor (P) and contains the signal peptide (SP) from the *apf* gene and a translational stop codon (pushpin). Plasmid pAF100-VHHA6 allows production and secretion of soluble VHHA6 with a fused C-tag. (**b**) Soluble VHH (14.4 kDa) was detected in the supernatant from *L. rhamnosus* DSM 14870 pAF100-VHHA6 by Western blot using a biotinylated camelid anti C-tag antibody and streptavidin-HRP via ECL. pIA7: empty vector control, SN: supernatant, CE: cell extract (**c**) Neutralizing activity of soluble VHHA6 expressed in *E. coli* or *L. rhamnosus* pAF100-VHHA6 using a panel of HIV-1 pseudoviruses. Standardized TZM-bl assays were performed to determine IC_50_ values [μg/mL]. The panel of pseudoviruses includes the most common subtypes B and C and patient relevant Tier 2 and 3 neutralization sensitivities. IC_50_ values are colour-coded (>50 μg/mL: white, 10–50 μg/mL: yellow, 1–10 μg/mL: orange, 0.1–1 μg/mL: red, <0.1 μg/mL: dark red) and were determined from the respective neutralization curves using GraphPad Prism.

To obtain a self-replicating system expressing HIV-1-neutralizing nanobodies, the expression cassette for VHHA6 was cloned into the pIAV7 vector [50] generating the plasmid pAF100-VHHA6 (Figure 3a). This plasmid allows the production of soluble nanobodies from *L. rhamnosus*, which are detectable via an antibody against the C-terminally fused C-tag. The corresponding band of 14.4 kDa could be detected by Western blot in the culture supernatants of *L. rhamnosus* DSM 14870 pAF100 VHHA6 using a camelid anti-C-tag antibody (Figure 3b). The neutralizing activity of C-tag purified VHHA6 from *L. rhamnosus* was compared to that of VHHA6 produced in *E. coli* in the TZM-bl assay using a representative panel of relevant HIV-1 pseudoviruses of different subtypes and neutralization sensitivities. The neutralizing capacity of nanobody VHHA6 expressed from *L. rhamnosus* was maintained for all pseudoviruses tested and was in a similar range, as observed for the respective protein produced in E. coli (Figure 3c).

#### 3.2.2. Expression of VHHA6 Anchored on the Surface of *L. rhamnosus* DSM 14870 pAF900-VHHA6

**Figure 4:**
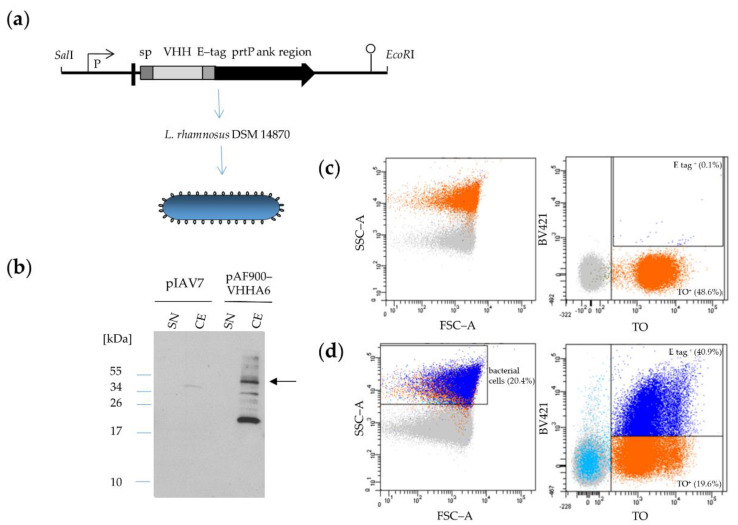
Expression of cell-associated VHHA6 from *Lactobacillus rhamnosus* DSM 14870-pAF900-VHHA6. (**a**) The expression cassette was cloned into the pIAV7 plasmid [50] between SalI and EcoRI restriction sites generating the plasmid pAF900-VHHA6. The expression cassette of VHHA6 is controlled by the *apf* promotor (P) and contains the signal peptide (SP) from the *apf* gene and a translational stop codon (pushpin). Plasmid pAF900-VHHA6 allows production of cell wall attached VHHA6 with a fused E-tag. Cell wall anchoring is mediated via a prtP anchor (ank) region fused to the VHH. (**b**) VHHA6 (40 kDa) was detected by Western Blot in the cell extract using an anti-E-tag antibody (arrow). pIAV7: empty vector control, SN: supernatant, CE: cell extract. (**c**,**d**) Surface display of VHHA6 was shown by flow cytometry. *L. rhamnosus* DSM 14870 were stained with Thiazole Orange (TO), whereas VHHA6 was detected via its E-tag with rabbit α-E tag and goat α-rabbit IgG-BV421 (BV421). The dark blue population is double positive for TO and the E-tag associated to VHH. Background signal with wildtype bacteria was 0.1% (**c**, right upper panel), while 41% of pAF900-VHHA6 bacteria showed VHHA6 expression on the surface (**d**, right upper panel). Light blue population represents unstained bacteria (for TO), whereas non-bacterial particles are shown in grey.

Besides VHHA6 expressed in a soluble form, we also tested VHHA6 expressed in a cell-wall-anchored form linked to the *prtP* anchor region on the surface of *L. rhamnosus* cells (Figure 4). VHHA6 could be detected in the corresponding bacterial cell extract as a 40 kDa protein via Western blot based on the E-tag at its C-terminus (Figure 4b). The presence of bands at higher molecular size is most likely due to peptidoglycan subunits covalently linked to the recombinant protein which retard its migration, while the bands at lower molecular weight are degradation products within the bacterial cells due to overexpression of the recombinant protein [56]. We further verified expression of VHHA6 on bacterial cells by flow cytometry (Figure 4c,d). *L. rhamnosus* DSM 14870 were stained with Thiazole Orange, which incorporates into nucleic acids [57], whereas VHHA6 expression on the surface was detected through its E-tag using goat α-rabbit IgG BV421 against the rabbit α-E-tag Mab. About 41% of *L. rhamnosus* DSM 14870 pAF900-VHHA6 constitutively expressed VHHA6, whereas background staining of wildtype bacteria was 0.1%. After fluorescence activated cell sorting of E-tag positive bacteria transformed with pAF900-VHHA6, the expression of VHHA6 was analyzed over 4 days by inoculation into MRS medium with erythromycin every 24 h. The detection/expression of VHHA6 could be enriched by cell sorting, but decreased over time after daily reinocculations, until it reached the level of unsorted pAF900-VHHA6 bacteria again after 65 h (Supplementary Figure S2).

##### Lactobacilli with Surface-Anchored VHHA6 Bind GFP-Labeled HIV-1 Virions

Next, we wanted to analyze the binding of GFP labeled HIV-1 virions to *L. rhamnosus* DSM 14870 pAF900-VHHA6 expressing nanobody VHHA6 on the surface by flow cytometry. To this end, we incubated bacterial cells with Gag-eGFP pCHIV virus particles [52] and first ensured binding of VHHA6 to NL4-3 Env by ELISA using NL4-3 SOSIP proteins, as pCHIV is derived from this strain. Binding of VHHA6 to NL4-3 SOSIP was confirmed and comparable to binding to optC.664 SOSIP, used as positive control (Supplementary Figure S3).

In the flow cytometry binding assay of eGFP labeled virus particles to *L. rhamnosus* with surface-anchored VHHA6, we included several controls (Supplementary Figure S4): *L. rhamnosus* DSM 14870 transformed with empty vector (pIAV7, panels a–c) were compared to *L. rhamnosus* DSM 14870 expressing VHHA6 (pAF900-VHHA6, panels d–f) and binding of virus particles carrying Env on their surface (pCHIV + Env, panels c, f) was compared to Env-deficient virus particles (pCHIV–Env, panels b,e). We observed background binding of virus particles lacking Env to *L. rhamnosus* DSM 14870 with and without VHHA6 in the range of 7.2 to 8.0% (panels b and e). Interestingly, the incorporation of Env into tvirus particles (pCHIV + Env) reduced unspecific binding to non-expressor *L. rhamnosus* DSM 14870 (pIAV7) (2.3%, panel c) and enhanced specific binding to *L. rhamnosus* DSM 1487 with surface-displayed VHHA6 (pAF900-VHHA6) (12.4%, panel f).

**Figure 5:**
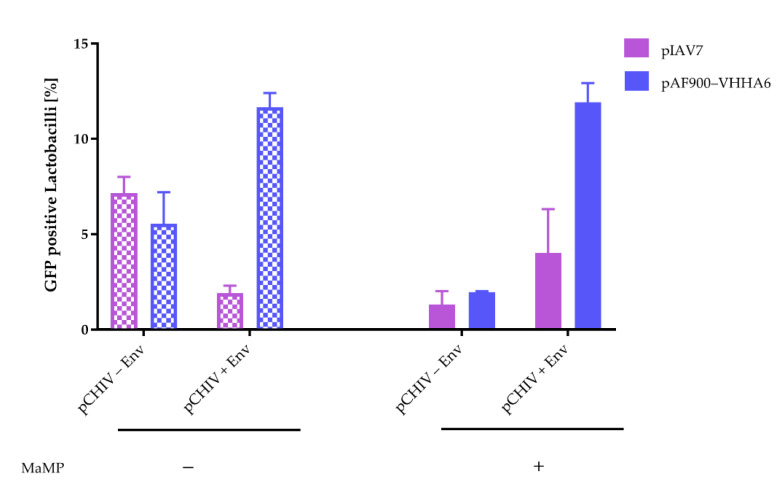
Reduction in background binding of GFP-labeled HIV-1 virus particles lacking Env to Lactobacilli by Methyl-α-D-mannopyranoside (MaMP) analyzed by flow cytometry. The addition of MaMP (0.05 M, right panel) reduces background binding of GFP-labeled HIV-1 virus particles lacking Env (pCHIV − Env) to L. rhamnosus DSM14870 expressing VHHA6 on the surface (pAF900-VHHA6, blue) or not (pIAV7, purple). MaMP has no effect on specific binding of NL4-3 viral particles with Env (pCHIV + Env) to lactobacilli expressing VHHA6 (blue: 12.4% compared to 11.9%) and only marginally increases binding to wildtype Lactobacilli (purple: 2.3% compared to 4.1%). Error bars represent SEM of two experiments.

Unspecific binding of pCHIV–Env particles could be further reduced by the addition of Methyl-α-D-mannopyranoside (MaMP), which interferes with the binding of glycoproteins to lectins and adhesion molecules on the surface of *L. rhamnosus* DSM 14870 (Figure 5) [58]. Binding to non-expressor *L. rhamnosus* DSM 14870 pIAV7 was reduced from 8.0% to 1.3%, binding to VHHA6 expressing *L. rhamnosus* DSM 14870 pAF900-VHHA6 from 7.2% to 2.0%. In contrast, MaMP had no effect on specific binding of pCHIV + Env virus particles to *L. rhamnosus* DSM 14870 expressing VHHA6 (pAF900-VHHA6: 12.4% vs. 11.9%) and only marginally increased binding to non-expressor *L. rhamnosus* DSM 14870 (pIAV7: 2.3% to 4.1%).

##### Lactobacilli with Surface-Anchored VHHA6 Neutralize HIV-1 in an Infectivity Depletion Assay

We then analyzed surface-anchored VHHA6 on *L. rhamnosus* DSM 14870 pAF900-VHHA6 for its ability to neutralize HIV-1. For this, we performed an infectivity depletion assay. In short, we incubated irradiated *L. rhamnosus* DSM 14870 pAF900-VHHA6 serially diluted in cell culture medium (in the presence or absence of MaMP) with a constant amount of pseudoviruses to allow for binding. After centrifugation, unbound infectious virus remaining in the supernantant was quantified by titration on TZM-bl indicator cells. The half-maximal inhibitory concentration (IOD_50_) of *L. rhamnosus* DSM 14870 pAF900-VHHA6 needed to deplete 50% of the infectivity in the supernatant was determined for a reference panel of different pseudovirus subtypes that displayed different neutralization sensitivities (Table 1, compare Figure 3c).

As observed before (Figure 4), the addition of MaMP strongly reduced unspecific binding of virus particles to *L. rhamnosus* DSM 14870, resulting in higher IOD_50_ being needed to deplete 50% of infectious pseudovirus from the supernatants, in particular for the non-expressor *L. rhamnosus* DSM 14870 pIAV7 (pIAV7). All pseudoviruses were neutralized with IOD_50_ values ranging from 0.27 to 2.54, whereas the IOD_50_ of the MLV control (4.48) is comparable to the IOD_50_ of the *L. rhamnosus* DSM 14870-pIAV7 control that does not express VHHA6 on the surface. These data clearly prove the functionality, i.e., the neutralization capacity, of VHHA6 anchored on the surface of *L. rhamnosus* DSM14870 pAF900-VHHA6. Furthermore, the neutralization strength was roughly comparable to the corresponding data with soluble VHHA6, CNE55 being the most difficult virus to neutralize by this nanobody.

## 4. Discussion

After the demonstration that bnAbs can prevent infection by HIV-1 in animal models, some of them have advanced to clinical trials of passive immunization both, in therapeutic and preventive settings [59,60]. Therapeutic application of some of the most effective bnAbs targeting the CD4 receptor-binding site in Env during ART interruptions, as monotherapy generally resulted in rapid development of resistance [4,5,8,10,21]. Thus, a combination of bnAbs targeting different Env epitopes, or even more efficient bnAbs that do not permit escape mutations of the virus, such as the recently identified mAb 1–18 [11], will be needed. Preventive trials in cohorts at high risk of HIV-1 transmission are ongoing (NCT02716675 and NCT02568215) [23].

Although very promising, the approach to use bnAbs for passive immunization studies also has several practical limitations. First, the bnAbs have to be present in the body in a constant high concentration of at least 10 µg/mL in order to protect from low-dose rectal viral challenges in the non-human primate model [59]. This requires infusions of 20–30 mg bnAb/kg body weight every two months due to the half-life of 11–24 days for IgG molecules [5,6,61]. Second, recombinant expression of IgG in gram quantities is expensive and sometimes problematic due to the intramolecular cysteine bridges needed for correct folding. Finally, i.v. application of antibodies has to be performed in a clinical setting. In contrast, vector-mediated expression could potentially prolong the intervals between applications, and AAV-derived vectors are currently being explored for bnAbs delivery [62,63,64,65]. However, antibodies mounted against the AAV-expressed therapeutic antibody have already been observed for these vectors in different settings [66,67]. For the prevention of heterosexual transmission, bnAbs should also be active at the primary site of transmission, the vagina. However, antibodies are unstable at the acidic environment in the healthy human vagina (pH 4–4.5, [30], see Figure 2). To overcome these limitations, nanobodies or VHHs derived from *Camelidae* may be better suited due to their small size (15 kDa, i.e., 1/10 of a conventional antibody) and favorable physicochemical properties (high solubility and stability) [26]. Interestingly, VHHs share the unconventional paratope structures often found in bnAbs against HIV-1 such as extra-long HCDR3 loops, and they have high homology to human VH3 genes [27].

We and others have previously selected potent bn VHHs targeting the HIV-1 Env protein, and preferentially the cavity representing the CD4-binding site [28,29]. In the present study, we could prove that one of our best bn nanobodies, VHHA6, can indeed protect against HIV-1 infection in a humanized mouse model. Because of the short half-life of the VHHs, nanobodies were preincubated with HIV-1_BAL_ prior to i.r. challenges of CD34T+ humanized mice (Figure 1). All mice (9/9) were protected from three consecutive i.r. challenges at days 0, 1 and 2 by HIV-1_BAL_ in the group receiving VHHA6. In contrast, 5/8 mice (62.5%) were infected in the control groups receiving either the challenge alone or preincubated with an unrelated control VHH (6G2), which is in line with the HIV-1_BAL_ transmission efficiency of 50–65% we observed in this mouse model. Thus, we can conclude that A6 is capable of preventing mucosal HIV-1 infection in vivo, qualifying it as a promising candidate for preventive applications. However, due to the short half-life of VHHs, which comes along with their small size, vector-mediated expression is needed to maintain a continuous effective concentration of bn VHHs at the sites, where HIV-1 transmission primarily occurs. VHHs are particularly suited for such an approach due to their low immunogenicity. Indeed, multiple injections of VHHs in mice, monkeys and patients did not show adverse effects [68,69,70,71,72,73]. To date, only one tetravalent agonistic VHH directed against death receptor 5 (DR5) showed transient hepatotoxicity in a clinical trial [74]; however, this was not due to the VHH per se, but rather to increased clustering of DR5 receptors resulting in apoptosis.

As our aim was to develop a protective passive vaccine against HIV-1, in particular for women in developing countries who may not always have optimal access to antiretroviral drugs with regard to HIV prevention, we chose lactobacilli as vectors to express bn VHHs due to their favorable properties, described above. Previous studies have already shown the potential of lactobacilli for delivery of microbicides against HIV-1/SHIV infection including antibody fragments [41,75], the chemokine RANTES [76], cyanovirin [77] and two-domain CD4 [36] in vitro and in animal models. Here we chose *L. rhamnosus* DSM 14870 as vector, because this strain is already a constituent of EcoVag^®^ vaginal capsules for treatment of bacterial vaginosis and has been shown to colonize the human vagina [45,47,48]. Furthermore, the genome of this strain has been completely sequenced and does not contain antibiotic resistance genes ensuring its safety for clinical applications [46]. Plasmid systems for the expression of soluble or surface-displayed antibody fragments have been previously developed in the Marcotte group, as well as a stable chromosomally integrated expression system [42]. Thus, *L. rhamnosus* DSM 14870 is particularly suited as vector for the expression of VHHA6.

We expressed VHHA6 from *L. rhamnosus* DSM 14870, both in a soluble and in a cell-wall-anchored form, which would easily allow to combine two different nanobodies for even broader coverage of HIV-1 strains. Soluble purified VHHA6 maintained its neutralizing activity in the TZM-bl assay against a small panel of HIV-1 pseudoviruses, representing the most prevalent HIV-1 subtypes C, B and A/E (Figure 3c). IC_50_ values were largely comparable to those derived in parallel for VHHA6 expressed from *E. coli*. Expression of cell wall-anchored VHHA6 was detected via the C-terminal E-tag by Western blot of bacterial cell lysates and by flow cytometry on the surface of lactobacilli (Figure 4). Despite selection with erythromycin, which is encoded in the pAF900-VHHA6 plasmid, the percentage of *L. rhamnosus* DSM-14870-expressing, surface-bound VHHA6 stabilized at approximately 30–40% (Figure 4d) and could not be further improved by repetitive inoculations of sorted cells (Supplementary Figure S2). Loss of expression of membrane-anchored proteins in lactobacilli due to plasmid instability was previously observed by us for other recombinant proteins, and integration of the expression cassettes into the *Lactobacillus* genome should be performed to stabilize expression [42]. Shutdown of protein expression from the plasmid or mutation in the LPXTG motif or anchoring system (sortase) of *PrtP* may also be reasons for the absence of transgenic proteins on the surface, which has yet to be tested.

We could show by flow cytometry analyses that HIV-1 NL4-3 virus particles covalently labeled with the GFP protein [52] indeed bound to *L. rhamnosus* DSM 14870 pAF900-VHHA6 with surface-anchored VHHA6 (Supplementary Figure S4f). Unspecific binding of virus particles to *L. rhamnosus* DSM 14870 pIAV7 not expressing VHHA6 was also observed to a lesser extent (Supplementary Figure S4c). This is probably mediated by the interaction of lectins on the surface of lactobacilli, which bind glycosylated proteins on pathogens like bacteria and viruses [78]. Interestingly, we also observed unspecific binding of HIV virus particles lacking Env to *L. rhamnosus,* independently of the expression of VHHA6 (Supplementary Figure S4b,e), which may be mediated by glycosylated cellular proteins incorporated into the viral membrane during budding. In line with this, we could reduce unspecific background by the addition of MaMP, a sugar also used to elute glycosylated viruses from lectin columns for virus purification [79] (Figure 5). This uncovered strong specific binding of GFP-labeled NL4-3 HIV-1 particles to the *L. rhamnosus* DSM 14870 pAF900-VHHA6 expressing surface-attached VHHA6.

We could further determine specific neutralizing activity of VHHA6 displayed on the surface of *L. rhamnosus* DSM 14870 pAF900-VHHA6 against a panel of representative HIV-1 pseudoviruses in our infectivity depletion assay (Table 1). This assay is a modification of the classical TZM-bl assay that we used for soluble VHHA6. To avoid the addition of *L. rhamnosus* DSM 14870 pAF900-VHHA6 with bound pseudoviruses to the TZM-bl cells, even though lactobacilli were irradiated, we instead determined the amount of unbound pseudovirus remaining in the supernatants of the bacterial cells for the various bacterial concentrations indicated as optical densities (OD_600_). Then, the IOD_50_ was determined, i.e., the *L. rhamnosus* DSM 14870 pAF900-VHHA6 concentration needed to deplete 50% of virus particles from the supernatants. We observed specific neutralization of HIV-1 pseudoviruses mediated by surface-attached VHHA6 (Table 1: IOD < 1 for 6/8 pseudoviruses for pAF900-VHHA6 compared to IOD_50_ > 3 for 7/8 in the control group pIAV7). As shown above, the addition of MaMP reduced unspecific binding to lectins and consequently neutralization of pseudoviruses by Lactobacilli without VHHA6 on the surface (pIAV7). Potentially, extracellular vesicles that are common to several bacterial strains including Gram-positive *L. rhamnosus* and have been shown to inhibit HIV-1 ex vivo may also contribute to neutralization [80,81].

## 5. Conclusions

In this study, we could demonstrate that the bn nanobody VHHA6 protects from mucosal HIV-1 infection in CD34T+ humanized mice and is still functional at acidic pH, as found in the healthy human vagina. Further, VHHA6 expressed from *L. rhamnosus* DSM 14870, both in a soluble and in a membrane-anchored form, neutralized primary HIV-1 strains of different subtypes in vitro. Thus, this *Lactobacillus* strain, represents a promising vector for passive vaginal immunizations in women at high risk of sexual HIV-1 transmission. Besides the antiviral activities intrinsic to Lactobacilli per se, this strain allows easy coexpression of bn VHHs targeting different epitopes in HIV-1 Env in a soluble and/or in a cell-wall-anchored form, which should mediate broad protection and minimize viral escape. Such preventive products are inexpensive to produce, stable at higher temperatures and easy to applicate at home and thus very promising for developing countries. Further, we have previously shown that *L. rhamnosus* DSM 14870 could transiently colonize the vagina of women and that weekly administration could increase the persistence of the bacteria on mucosal surfaces [47,48]. It is thus likely that vaginal capsules containing the recombinant lactobacilli would need to be administered on a regular basis to maintain their population level. However, lactobacilli-expressing VHHs are genetically modified organisms, and one has to restrict their unintended release into the environment. Chromosomal integration of the expression cassette and biological containment systems are therefore needed and are currently being developed, i.e., the recently described system of inducible plasmid self-destruction developed by the Marcotte group [82]. Our findings encourage us to propose the practical application of *L. rhamnosus* DSM 14870 as vector for prevention of mucosal HIV-1 infections in the near future. We are now planning to test the efficacy of the *Lactobacillus* producing VHH against HIV-1 in animal models.

## Figures and Tables

**Table 1:** Neutralization of HIV-1 by lactobacilli with surface-anchored VHHA6 (*L. rhamnosus*. pAF900-VHHA6) in the infectivity depletion assay.

			IOD_50_				
			−MaMP		+MaMP		
Pseudovirus	Tier	Clade	pIAV7	pAF900-VHHA6	pIAV7	pAF900-VHHA6	
ZM197	1B	C	1.43	0.643	5.00	0.274	IOD_50_ [OD_600_/well]
X2278-C2_B6	2	B	2.83	0.683	3.95	0.846	
TRO.11	2	B	4.41	0.637	4.95	0.551	
CE1178_A3	2	C	2.90	0.512	3.20	0.744	<1
HIV_25710_2.43	2	C	2.74	0.520	2.06	0.468	1–2.5
optC	n.d.	C	3.13	0.760	5.00	1.73	2.5–5
CNE55	2	CRF01_AE	-	-	3.52	2.54	
TRJO.4551	3	B	1.65	1.25	3.04	0.935	
MLV	-	-	4.87	4.99	4.38	4.48	

IOD_50_: half-maximal inhibitory concentration (measured as OD_600_) of pAF900-VHHA6 needed to deplete 50% of the pseudovirus infectivity in the supernatants.

## Supplementary Materials

The following are available online at https://www.mdpi.com/2076-393X/8/4/758/s1, Figure S1: Comparison of in vivo stability of antibody 3BNC117, heavy-chain only antibody VHHA6-Fc and soluble nanobody VHHA6 in mice, Figure S2: Loss of membrane-bound VHH expression from *L. rhamnosus* DSM14870 pAF900-VHHA6, Figure S3: Binding analysis of VHHA6 to NL4-3 SOSIP, Figure S4: Detection of binding of GFP-labeled HIV-1 virus particles to lactobacilli by flow cytometry.
