# Supplementary material for: Lactobacilli Expressing Broadly Neutralizing Nanobodies against HIV-1 as Potential Vectors for HIV-1 Prophylaxis?
Source: Vaccines (Basel). 2020 Dec 13;8(4):758. doi: 10.3390/vaccines8040758 (PMC7768517; doi:10.3390/vaccines8040758)
Supplement: Supplementary file 1 [file vaccines-08-00758-s001.pdf]

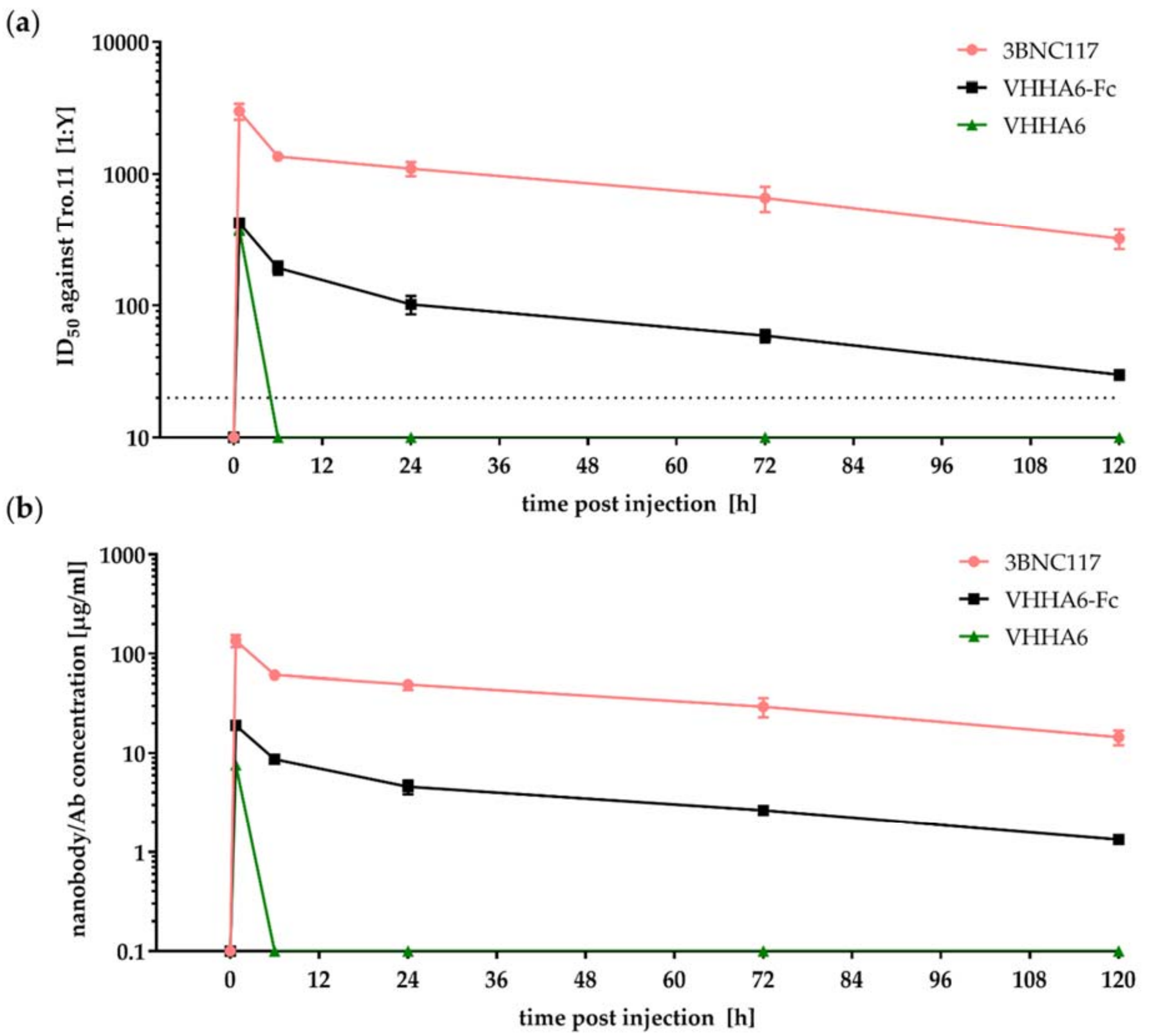

Figure S1. Comparison of *in vivo* stability of antibody 3BNC117, heavy-chain only antibody VHHA6-Fc and soluble nanobody VHHA6 in mice. The indicated proteins (500 µg in 200 µL PBS) were injected into the tail vein of uninfected NRG mice. Blood samples were taken at 0, 0.75, 6, 24, 72 and 120 hours post injection. (**a**) The half maximal inhibitory dose (ID_{50}) of serum dilutions (titer) neutralizing HIV-1 Tro.11 pseudovirus (clade B, tier 2) in the TZM-bl assay is shown for 3 animals per antibody/nanobody over time. (**b**) The concentration of antibodies/nanobodies in the sera was calculated based on the ID_{50} and IC_{50} values (Monoclonal IC_{50} values against HIV-1 Tro.11 were as follows: VHHA6: 0.020 µg/mL, VHHA6-Fc: 0.045 µg/mL, 3BNC117: 0.045 µg/mL). Error bars represent SEM of 3 mice.

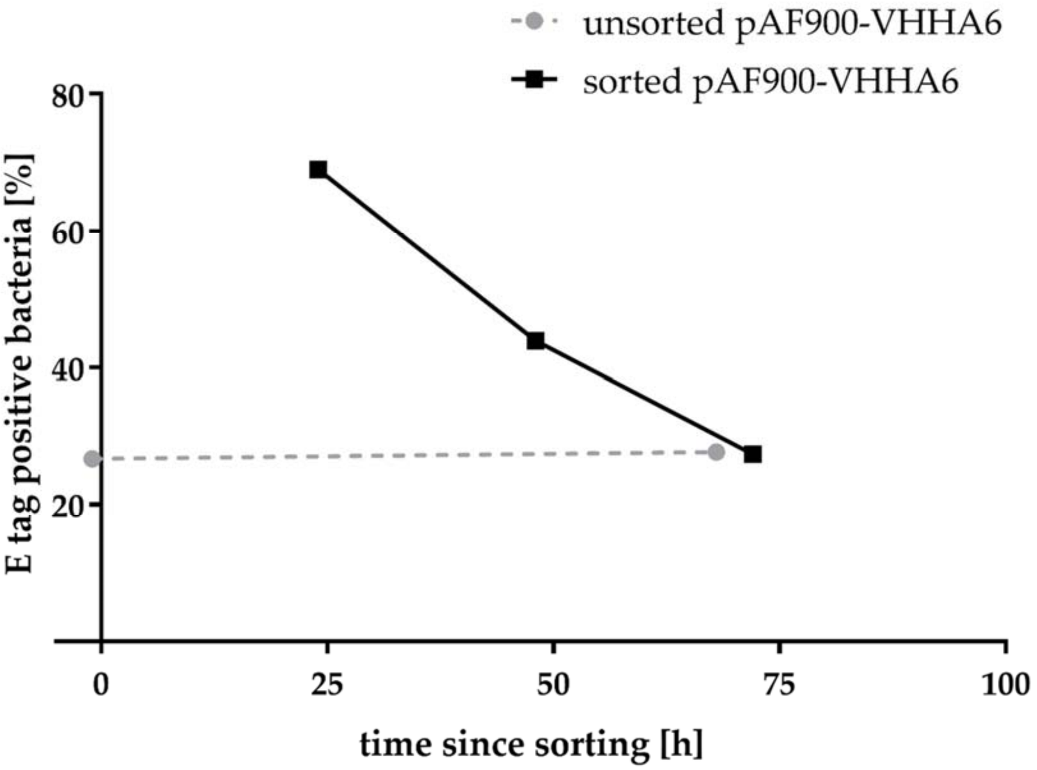

Figure S2. Loss of membrane-bound VHH expression from *L. rhamnosus* DSM14870 pAF900-VHHA6. After FACS sorting, E-tag positive cells were collected in MRS broth media with erythromycin for cultivation or storage at -80°C (after mixing 1:1 with 50% glycerol in PBS). Cultured cells were analyzed again by FACS as for unsorted cells. Surface expression of VHHA6 declined over time to reach again the expression level before sorting. FACS measurements of cultured sorted cells are indicated by black squares, those for uncultured cells are indicated by grey circles.

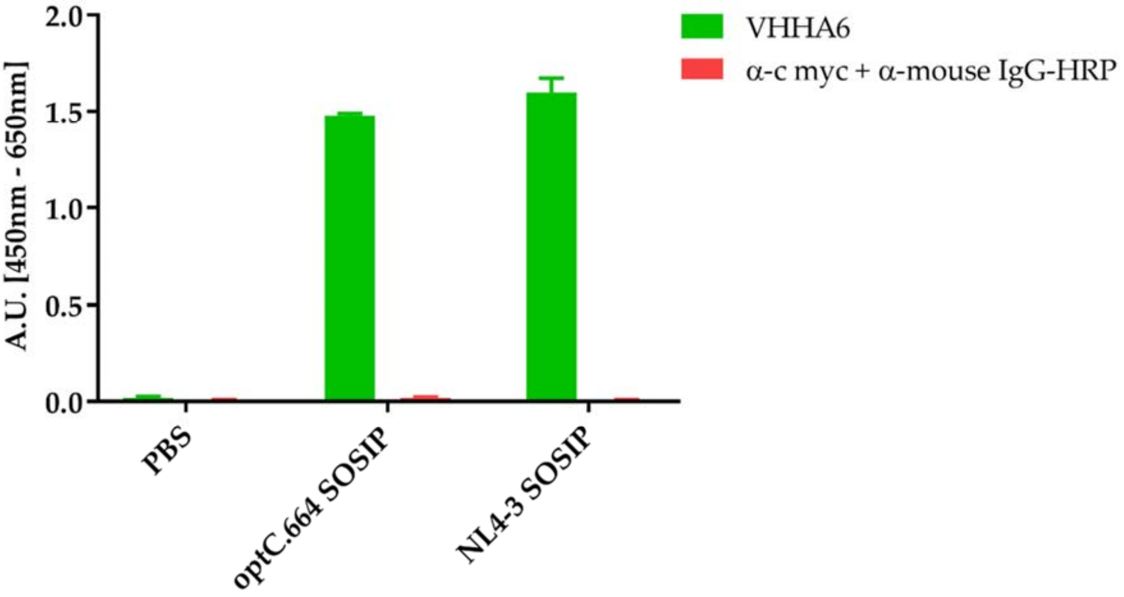

Figure S3. Binding analysis of VHHA6 to NL4-3 SOSIP. Binding of purified soluble VHHA6 to optC.664 and NL4-3 gp140 SOSIPs by ELISA. Binding experiments were performed using 100 ng VHH A6 and 200 ng of the immobilized gp140 SOSIPs. Binding was detected via the C-terminal myc-tag incorporated into the VHH protein. Error bars represent SEM of duplicates.

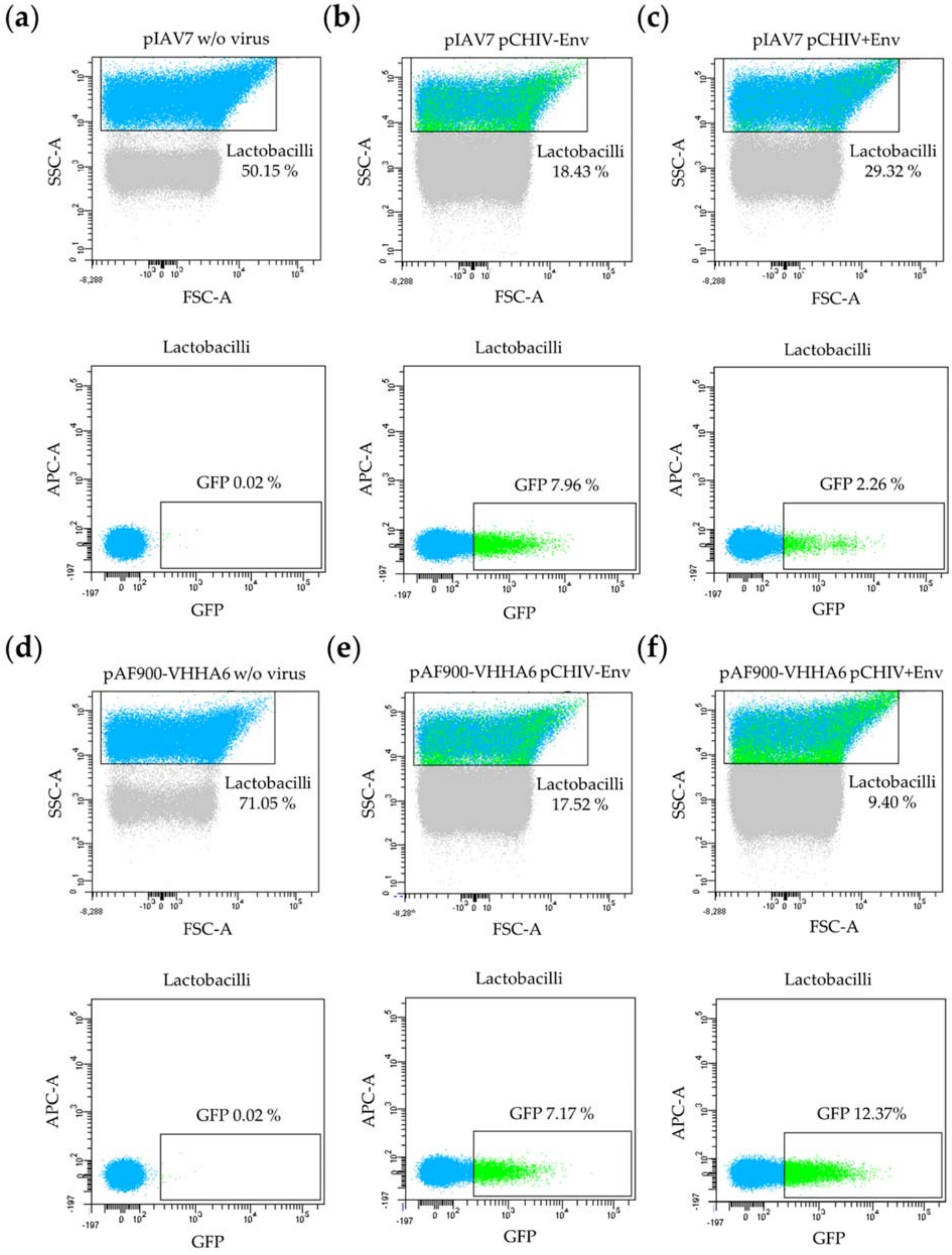
(a)
pIAV7 w/o virus
SSC-A
FSC-A
Lactobacilli
50.15 %
Lactobacilli
APC-A
GFP
GFP 0.02 %
(b)
pIAV7 pCHIV-Env
SSC-A
FSC-A
Lactobacilli
18.43 %
Lactobacilli
APC-A
GFP
GFP 7.96 %
(c)
pIAV7 pCHIV+Env
SSC-A
FSC-A
Lactobacilli
29.32 %
Lactobacilli
APC-A
GFP
GFP 2.26 %
(d)
pAF900-VHHA6 w/o virus
SSC-A
FSC-A
Lactobacilli
71.05 %
Lactobacilli
APC-A
GFP
GFP 0.02 %
(e)
pAF900-VHHA6 pCHIV-Env
SSC-A
FSC-A
Lactobacilli
17.52 %
Lactobacilli
APC-A
GFP
GFP 7.17 %
(f)
pAF900-VHHA6 pCHIV+Env
SSC-A
FSC-A
Lactobacilli
9.40 %
Lactobacilli
APC-A
GFP
GFP 12.37%

Figure S4. Detection of binding of GFP-labeled HIV-1 virus particles to Lactobacilli expressing or not VHHA6 by flow cytometry. For analysis 10 µl of eGFP-labeled (green) viral particles (50,000 events each, [Lampe, 2007]) were added to $3x10^7$ bacterial cells (light blue) for 5 hours. Upper panels: Non-expressor *L. rhamnosus* DSM14870-pIAV7 (pIAV7) **(a)** without viral particles (0.0% GFP positive), **(b)** with eGFP labled viral particles either lacking Env (8.0% GFP positive) or **(c)** with NL4-3 Env (2.3% GFP positive). Lower panels: *L. rhamnosus* DSM14870-pAF900-VHHA6 expressing surface anchored VHHA6 (pAF900-VHHA6) **(d)** without viral particles (0% GFP positive), **(e)** with eGFP labeled viral particles either lacking Env (7.2% GFP positive) or **(f)** with NL4-3 Env (12.4% GFP positive).
